# The safety and efficacy of a protein-free diet with ketoacid analogues in chronic kidney disease-affected diabetic rats

**DOI:** 10.1016/j.heliyon.2024.e41607

**Published:** 2025-01-10

**Authors:** Ahmed El-Sayed Nour El-Deen, Adel shallaby, Abdel Kader Ibrahim, Mohammed Abdel Aziz Mohammed, Ahmad Taha, Mohamed Zaeim Hafez Ahmed, Muhammad Abdelbaeth Elfiky, Ahmed A. Abd El-Rhman, Ahmed F. Abdel Ghany, Almoatazbellah Mahmoud Elsayed, Ahmed Noaman Ali, Ali Abdeslam

**Affiliations:** a Department of Physiology, Faculty of Medicine, Al-Azhar University, Assiut, Egypt; b Department of Basic Medical and Dental Sciences, Faculty of Dentistry, Zarqa University, Zarqa, Jordan; c Department of Physiology, Faculty of Medicine, Al-Azhar University, Cairo, Egypt; d Department of physiology, faculty of medicine Port Said University, Egypt; e Department of Pathology, Faculty of Medicine, Al-Azhar University, Assiut, Egypt; f Department of Oral pathology department, Faculty of Dentistry, Tanta University, Egypt; g Department of pharmacology, Faculty of Medicine, Al-Azhar University, Assiut, Egypt

**Keywords:** Diabetes, Kidney disease, Protein diet, Ketoacid and protein-free diet

## Abstract

**Background:**

Diabetic nephropathy (DN) is a common vascular complication of diabetes Miletus (DM) that require weight control and dietary restrictions, especially for protein. A protein-restricted diet with ketoacid analogs (KA) reduces the intake of nitrogen while avoiding the harmful consequences of inadequate dietary protein intake.

**Objective:**

This study aimed to investigate the efficacy and safety of a protein-free diet with ketoacid analogs in chronic kidney disease (CKD)-affected diabetic rats.

**Material and methods:**

Sixty adult male albino rats were grouped into six equal groups (G): G (1) Control group, G (2) Normal rats. Received low protein diet, G (3) Diabetic control rats. Received standard diet, G (4) Diabetic rats. Received low protein diet for 12 weeks, G (5) Diabetic received α-keto amino acids (KAA) with a low protein diet and G (6) Diabetic received α-keto amino acids with protein-free diet. Blood sample was used to assess blood glucose (mg/dl), insulin (pmol/L), urea (mg/dl), creatinine (mg/dl), total cholesterol (TC) (mg/dl), LDL (mg/dl), HDL (mg/dl), triglycerides (TG) (mg/dl), and albumin (mg/dl) levels.

**Results:**

A significant decrease in blood glucose, serum total cholesterol, LDL, triglycerides, serum urea and creatinine were observed while insulin level, albumin, glomerular filtration rate (GFR), urine volume and HDL were significantly increased in group six.

**Conclusion:**

A protein-free diet containing KAA improves renal function, lowers blood glucose levels, maintains body weight, and does not worsen nutritional status in DN over time.

## 1 Introduction

Diabetes mellitus (DM) is a syndrome characterized by hyperglycemia due to a deficiency in insulin secretion, action, or both. Damage, dysfunction, and failure of end organs such as the kidney, retina, heart, nervous system, and vasculature are all linked to chronic hyperglycemia [1]. Diabetes mellitus is one of the most prevalent chronic diseases that can lead to costly, life-threatening consequences [2]. Diabetic nephropathy (DN) is a common vascular complication of DM, it develops in both types 1 and 2 of diabetes in about 40 % of patients [3]. Diabetes mellitus is the leading cause of chronic kidney disease (CKD) worldwide, comprising approximately 30 % of patients with non-dialysis CKD and 30 %–50 % of patients reaching end-stage renal disease [2]. Nutritional management with dietary protein restriction in addition to optimal glucose, lipid, proteinuria, and blood pressure (BP) control, is a central component of conservative treatment for patients with non-dialysis diabetic kidney disease (DKD) [3]. The prevention and management of chronic complications in individuals with any type of diabetes focused on nephropathy, retinopathy, neuropathy, and atherosclerotic cardiovascular disease (ASCVD) [4]. The pathological events that lead to DKD are caused by the hemodynamic as well as the metabolic factors that have the ability to activate intracellular signaling, hypoxia and finally lead to kidney inflammation and fibrosis [3].

DKD is usually diagnosed, clinically, by albuminuria or a lower glomerular filtration rate (GFR), while renal biopsy not required [5]. It is recommended to screen for CKD for patients diagnosed over 5 years with type 1 DM and in all patients with type 2 DM [4]. Patients with an albumin-to-creatinine ratio (ACR) of more than 30 mg/g creatinine, and/or an eGFR of less than 60 mL/min/1.73 m^2^ should be evaluated for CKD twice a year [6]. Calculation of the amount of calorie intake from lipids, carbohydrates, protein and salts is essential for body weight management, blood glucose level control, and decreasing diabetic vascular complications, especially DN. During the management of DN, weight control and dietary restriction especially for protein is crucial [4]. Accurate evaluation of the ideal amount of protein intake given its benefits and risks is of utmost importance [7]. A low-protein diet (LPD) has many benefits for patients with CKD by reducing nitrogen waste products and decreasing renal workload [8]. The LPD decreases intraglomerular pressure, which has a kidney protective effect, especially in patients with a decreased functioning nephron [9].

Ketoacid analogs (KA) of essential amino acids serve as substrates for protein synthesis without the production of toxic nitrogenous waste products. One of the highly restricted LPD for CKD uses amino acids and ketoacids as supplements (LPD-KA) to meet the body's bare minimum nitrogen requirements and reduce the nitrogen load [10]. LPD-KA decreases the intake of nitrogen while avoiding the deleterious consequences of inadequate dietary protein intake [11]. The benefits and harms of KA supplementation to protein-restricted diets in DKD are still in trials and the main risk is malnutrition [3].

The aim of this study was to investigate the efficacy and safety of a protein-free diet with ketoacidosis in CKD-affected diabetic rats.

## 2 Materials and methods

### 2.1 Ethical approval

The study was approved on December 11, 2019 by the Ethics of Scientific Research and the Use of Laboratory Animals Committee at Al-Azhar University, Egypt.

### 2.2 Experimental animals

Animal models used in this study were 60 male albino rats, with initial body weights ranging from 150 to 200 g (6–8 weeks old). Rats were purchased from the animal house at Assiut University in Egypt's Faculty of Medicine. Then held for two weeks as acclimation period in the Pharmacology Department of the Faculty of Medicine at Al Azhar University (Assiut branch). The animals were kept under controlled room temperature with a 12-h light-dark cycle with ad libitum access to food and water in conventional polypropylene cages with stainless steel hardware, Rats were divided into six equal groups, designated as follows:

Group 1 Normal control. Received standard diet.

Group 2 Normal rats. Received low protein diet.

Group 3 Diabetic control rats. Received standard diet

Group 4 Diabetic rats. Received low protein diet for 12 weeks.

Group 5 Diabetic rats. Received α-keto amino acids with a low protein diet for 12 weeks.

Group 6 Diabetic rats. Received α-keto amino acids with a protein-free diet for 12 weeks.

The procedure was continued for 12 weeks.

### 2.3 Induction of type 2 DM

The rats were fed a high-fat diet for two weeks, followed by a single intraperitoneal injection of 35 mg/kg body weight streptozotocin dissolved in citrate buffer pH 4.5 [12].

After receiving the injection of streptozotocin, the rats were maintained on a 10 % oral glucose solution together with their chow for the next 48 h [13].

The purpose of administering glucose was to prevent hypoglycemia since STZ can cause lethal hypoglycemia due to the death of beta cells, which triggers a large-scale release of pancreatic insulin [13].

The rats were tested for serum glucose levels using an Accu-Chek glucometer from Roche (Germany) seven days after the injection. After 2 h of glucose ingestion, rats were deemed diabetic if their serum glucose levels were above 250 mg/dl [14].

### 2.4 Experimental diets

The composition of the experimental diets were shown in Table (1)**.** These diets were designed according to the American Institute of Nutrition Rodent Diets (AINRD). Values are expressed as grams of a constituent per 100 g of diet. The protein composition of the various diets was calculated using casein's protein content (81.1 %) [[15], [16], [17]].

**Table 1:** Shows the composition of the experimental diets.

	Free protein diet	Low protein diet (8 %)	Normal protein diet (17 %)
1. Corn starch.	73.59	63.15	51.90
2. Casein.	0.00	9.9	21
3. Sucrose.	11.9	10	10
4. Soybean oil.	4.80	7	7
5. Fiber.	5	5.0	5
6. Minerals.	3.50	3.5	3.5
7. Vitamins.	1	1.0	1
8. L-cystine.	0.31	0.3	0.31
9. Choline bitartrate.	0.250	0.25	0.250
10. Tert-butylhydroquinone.	0.0015	0.0015	0.0015

**Alpha keto amino acids**: 0.125 g/kg body weight daily as a supplement.

The level of blood glucose was measured daily.

#### 2.4.1 Body weight measurement

For all the rats, the body weight (BW) was assessed once per week using a weighing scale (Ohaus model, 110 III–III L USA)

### 2.5 Collection of blood samples

After 12 weeks, the rats were sacrificed, and blood samples were obtained from the carotid artery. The blood was transferred into plain blood collecting tubes, allowed to clot and then centrifuged at 2500 rpm for 15 min. Subsequently, serum was collected and stored at −80 °C until used.

The stored serum sample was used to assess blood glucose (mg/dl), insulin (pmol/L), urea (mg/dl), creatinine (mg/dl), total cholesterol (TC) (mg/dl), LDL (mg/dl), HDL (mg/dl), triglycerides (TG) (mg/dl), and albumin (mg/dl) levels.

### 2.6 Collection of urine samples

The rats were placed in a metabolic cage for collection of urine from 8 a.m. to 8 a.m. of the next day for assessment of 24-h proteins in urine by centrifugation.

### 2.7 Histopathological examination

After sacrificing the rats, the abdomen was opened, and the kidney and pancreas were removed. The pancreas specimens were fixed immediately in 10 % neutral buffered formalin, embedded in paraffin, prepared as (5 mm) thick sections, and stained with Hematoxylin and Eosin (H&E) to assess the histopathological changes using the histological technique.

### 2.8 Statistical analysis

The statistical package for social sciences (SPSS, Version 22) was used to conduct the statistical analysis. Values were presented as means ± SE of the mean. One-way analysis of variance (ANOVA) was used to compare data from various groups. LSD test *post hoc* analysis of group differences was conducted. P values less than 0.05 were regarded as statistically significant.

## 3 Results

### 3.1 Low and protein-free diet decreases the final body weight level in diabetic rats

Our results revealed that the effect of ketoacids supplement on the final body weight (Per gram) was significantly higher in Group V and Group VI when compared with Group III and Group IV as shown in Table (2).

**Table (2):** Biochemical parameter of all groups.

	G1	G2	G3	G4	G5	G6	P Value
Final BW (Per grams)	215 ± 15	180 ± 10 ∗	181 ± 10	182 ± 11	195 ± 10@	197 ± 12 @	P < 0.0001
BG level (mg/dl)	116.9 ± 11.5	130.1 ± 12.7	250 ± 12.7∗	240 ± 3.4 ∗$	220 ± 2.3 ∗$@	205 ± 3. 7 ∗@^	P < 0.0001
Insulin Level (Uu/ml).	4.29 ± 0.71	4.45 ± 0.69	3.02 ± 0.731∗	3.01 ± 0.785∗	3.13 ± 0.712∗	3.03 ± 0.712∗	P < 0.0001
Creatinine (mg/dl).	0.45 ± 0.081	0.48 ± 0.076	1.0 ± 0.102∗	0.81 ± 0.149∗$	0.81 ± 0.149∗$@	0.69 ± 0.118∗@^	P < 0.0001
(BUN) (mg/dl)	12.36 ± 3.71	13.7 ± 3.93	21.8 ± 3.99∗	19.41 ± 4.19∗$	18.55 ± 3.86∗$@	16.55 ± 3.86∗@^	P < 0.0001
Urinary Proteins (g/24 h)	17.56 ± 4.37	18.22 ± 4.71	84.3 ± 3.6∗	76.8 ± 4.5∗$	70.8 ± 4.5∗$@	67.8 ± 4.5∗@^	P < 0.0001
Albumin (g/dl)	5.41 ± 0.588	5.38 ± 0.583	1.38 ± 0.53∗	2.2 ± 0.583∗$	3.38 ± 0.583∗$@	4.7 ± 0.5∗@^	P < 0.0001
Cholesterol (mg/dl)	88.4 ± 1.8	90.65 ± 2.5	125.6 ± 8.37∗	109.7 ± 6.4∗$	105.6 ± 8.37∗$@	100 0.6 ± 8.37∗@^	P < 0.0001
Triglycerides (mg/dl)	81.5 ± 2.7	83.6 ± 3.8	98.6 ± 3.8∗	92.5 ± 3.4 ∗$	86.7 ± 2.37 ∗$@	84.4 ± 2.37 ∗@^	P < 0.0001
LDL (mg/dl)	51.3 ± 11.6	55.3 ± 11.6	70.37 ± 11.6∗	67.5 ± 3.4 ∗$	63.7 ± 2.3 ∗$@	59.4 ± 2. 7 ∗@^	P < 0.0001
(HDL) (mg/dl)	65.3 ± 1.6	62.3 ± 11.6	49.37 ± 1.6 ∗	53.5 ± 3.4 ∗$	57.7 ± 2.3 ∗$@	60.4 ± 2. 7 ∗@^	P < 0.0001

All results are presented as means with standard deviations, one-way ANOVA with Tukey post hoc test (significance at p ≤ 0.05), ∗ Significant as compared to the control group, $ Significant as compared to Group III, @ Significant as compared with Group III and Group IV, ^ Significant as compared to Group III, Group IV and Group V.

### 3.2 Low and protein-free diet decreases the blood glucose levels in diabetic rats

The blood glucose levels decreased in groups (Group IV (from 49.37 ± 1.6 to 240 ± 3.4), Group V (from 250 ± 12.7 to 220 ± 2.3) and Group VI (from 250 ± 12.7 to 205 ± 3. 7) as mentioned in Table (2).

### 3.3 Low and protein-free diet decreases the serum creatinine level in diabetic rats

The serum creatinine declined in the following studied groups: Group IV (from 1.0 ± 0.102 to 0.81 ± 0.149), Group V (from 1.0 ± 0.102 to 0.81 ± 0.149) and Group VI (from 1.0 ± 0.102 to 0.69 ± 0.118). as mentioned in Table (2).

### 3.4 Low and protein-free diet decreases serum urea level in diabetic rats

Serum urea levels also declined in the following groups: Group IV (from 21.8 ± 3.99 to 19.41 ± 4.19), Group V (from 21.8 ± 3.99 to 18.55 ± 3.86) and Group VI (from 21.8 ± 3.99 to 16.55 ± 3.86). as mentioned in Table (2).

### 3.5 Low and protein-free diet increases serum albumin level in diabetic rats

In Group IV, serum albumin levels increased significantly. (from 1.38 ± 0.53 to 2.2 ± 0.583), Group V (from 1.38 ± 0.53 to 3.38 ± 0.583) and Group VI (from 1.38 ± 0.53 to 4.7 ± 0.5) and a decrease in proteinuria in Group IV (from 84.3 ± 3.6 to 76.8 ± 4.5), Group V (from 84.3 ± 3.6 to 70.8 ± 4.5) and Group VI (from 84.3 ± 3.6 to 67.8 ± 4.5). as mentioned in Table (2).

### 3.6 Low and protein-free diet improve the lipid profile in diabetic rats

A lipid spectrum analysis revealed Group IV to have lower total cholesterol. (from 125.6 ± 8.37 to 109.7 ± 6.4), Group V (from 125.6 ± 8.37 to 105.6 ± 8.37) and Group VI (from 125.6 ± 8.37 to 100.6 ± 8.37) and low-density lipoprotein in Group IV (from 70.37 ± 11.6 to 67.5 ± 3.4), Group V (from 70.37 ± 11.6 to 63.7 ± 2.3) and Group VI (from 70.37 ± 11.6 to 59.4 ± 2. 7). Serum triglyceride levels in Group IV decreased (from 98.6 ± 3.8 to 92.5 ± 3.4), Group V (from 98.6 ± 3.8 to 86.7 ± 2.37) and Group VI (from 98.6 ± 3.8 to 84.4 ±), Group V (from 98.6 ± 3.8 to 57.7 ± 2.3) and Group VI (from 98.6 ± 3.8 to 60.4 ± 2. 7). Also, there was an increase in the serum high density lipoproteins in Group IV (from 49.37 ± 1.6 to 53.5 ± 3.4), Group V (from 49.37 ± 1.6 to 57.7 ± 2.3) and Group VI (from 49.37 ± 1.6 to 60.4 ± 2. 7). as mentioned in Table (2).

## 4 Histopathological analysis

### 4.1 Pancreatic tissue

**Fig. 1:**
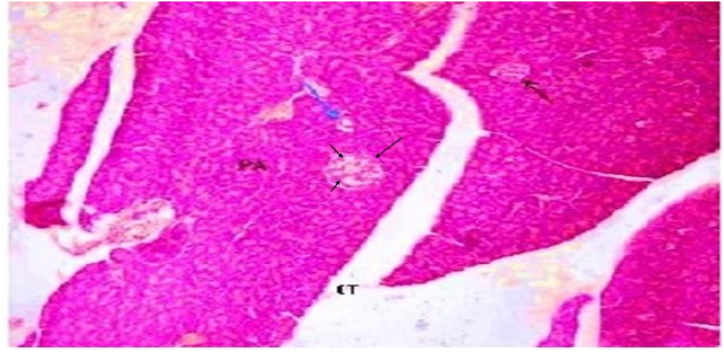
(A) A Photomicrograph of rat pancreatic tissue of the control group showing normal lobular architecture. Islets of Langerhans (black arrow), seen surrounded by the pancreatic acini (PA). Notice the Interlobular connective tissue (CT) and the interlobular duct (blue arrow). (Hx & E x 100).

The pancreatic tissue of rats in Group 1 that received a standard diet demonstrated normal acini that surround the Langerhans islets as shown in Fig. 1 and Table 3.

**Table: 3:** Histopathological results: Kidney.

	Acini	Renal cortex	Glomerular basement membrane	Mesangium
**Group 1**	Normal acini	Normal	No thickening	No mesangial enlargement
**Group 2**	No remarkable effect	Cords were created by islet's cells and divided by a web of blood capillaries	No thickening	No mesangial enlargement.
**Group 3**	Dilated inter-lobular duct with highly retained secretion in their pancreatic tissue.	Evidence of apoptotic cells with pyknotic nuclei in the vacuolated cells. nodular sclerosis	diffuse thickening of the glomerular basement membrane	Marked mesangial growth.
**Group 4**	No remarkable effect, but there is moderately congested blood capillaries around the pancreatic islets	Minimal inflammatory infiltrate within the islets	diffuse thickening of the glomerular basement membrane	Marked mesangial growth.
**Group 5**	Marked pancreatic duct dilation and a moderate amount of residual secretion. without nodular sclerosis as showed	No nodular sclerosis	Mild glomerular basement membrane thickening	Minor mesangial growth
**Group 6**	Mild dilatation of the pancreatic	Mildly congested blood capillaries around the pancreatic islets. No nodular sclerosis	No thickening	No mesangial enlargement

**Fig. 2:**
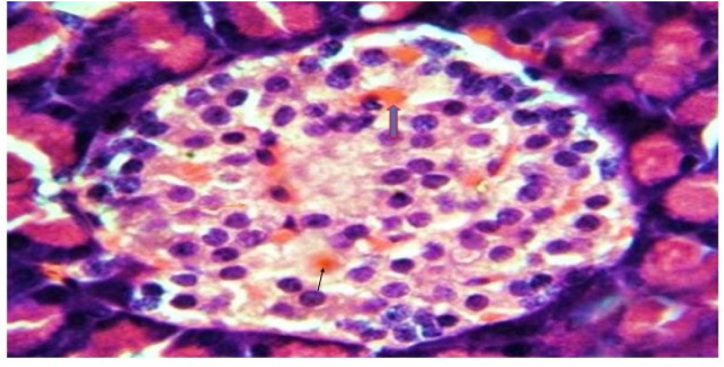
A photomicrograph of rat pancreatic tissue of the control group showing Islet's cells (blue arrow) forming cords separated by a network of blood capillaries (red arrow). Notice the pancreatic acini with its basal basophilia and apical acidophilia (A). (Hx&E x 1000).

In Group 2 with normal rats that received a low protein diet, cords were created by islet's cells and divided by a web of blood capillaries as mentioned in Fig. 2 and Table 3.

**Fig. 3:**
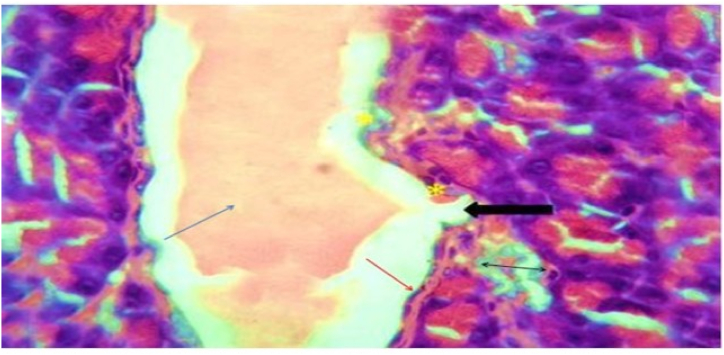
(A) Photomicrograph of a section in the rat pancreatic tissue of group III (Diabetic control received normal protein diet) showing highly dilated inter-lobular duct with highly retained secretion (blue arrow), lined with epithelial cells having flat nuclei (red arrow) while others have rounded one (∗). Notice the discontinuation of the epithelial lining (black arrow) and vacuolated cells with histological features of apoptotic cells with pyknotic nuclei (double arrow). (H&E x 400).

In Group 3 with diabetic control rats that received a standard diet exhibited greatly dilated inter-lobular duct with highly retained secretion in their pancreatic tissue with evidence of apoptotic cells with pyknotic nuclei in the vacuolated cells as displayed in Fig. 3 and Table 3.

**Fig. 4:**
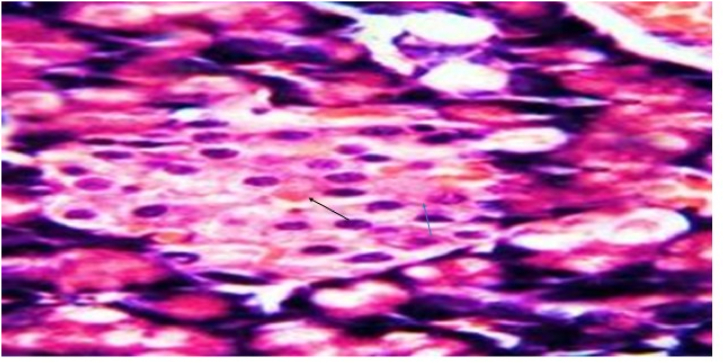
Photomicrograph of a section in the rat pancreatic tissue of group IV (Diabetic received low protein diet) showing moderate congested blood capillaries around the pancreatic islets (black arrows) and minimal inflammatory infiltrate within the islets (blue arrow). (Hx&E, ×1000).

In Group 4 Diabetic received a low protein diet showing moderately congested blood capillaries around the pancreatic islets and minimal inflammatory infiltrate within the islets as mentioned in Fig. 4 and Table 3.

**Fig. 5:**
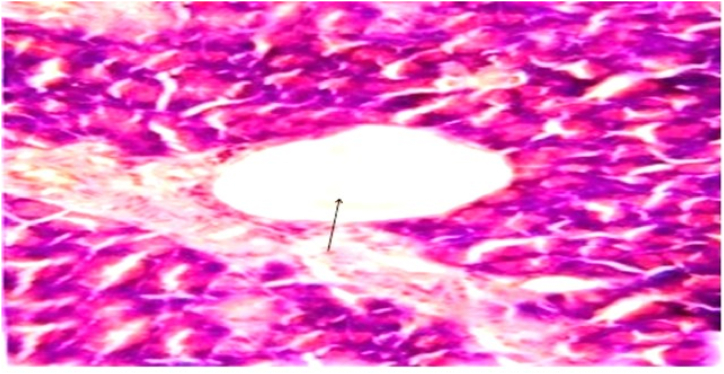
Photomicrograph of a section in the rat pancreatic tissue of group V (Diabetic received low protein diet) showing moderate dilatation of pancreatic duct with moderate retained secretion (black arrow). (H&E x 400).

In Group 5 rats with diabetes who received α-keto amino acids with a low protein diet for 12 weeks demonstrated a significant pancreatic duct dilation and a moderate amount of residual secretion as shown in Fig. 5 and Table 3.

**Fig. 6:**
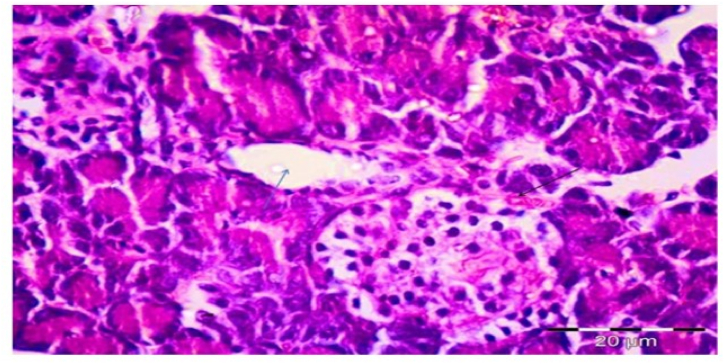
Photomicrograph of a section in the rat pancreatic tissue of group VI (Diabetic received α-keto amino acids with free protein diet) showing mild dilatation of pancreatic duct (blue arrow) and mild congested blood capillaries around the pancreatic islets (black arrows). (H&E x 400).

In Group 6 Diabetic rats received α-keto amino acids with a protein-free diet demonstrated mild dilatation of the pancreatic duct and mildly congested blood capillaries around the pancreatic islets as displayed in Fig. 6 and Table 3.

### 4.2 Renal tissue

**Fig. 7:**
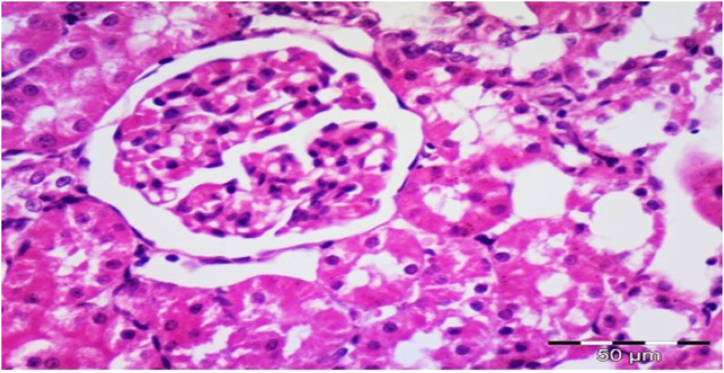
Photomicrograph of a section in the renal cortex of group I (control sham-operated) showing Malpighian renal corpuscle containing glomerulus without thickening of the glomerular basement membrane and no mesangial expansion (H&E, ×400).

The renal cortex revealed Malpighian renal corpuscle containing glomerulus with no thickening of the glomerular basement membrane and no mesangial enlargement as shown in Fig. 7 and Table 3.

**Fig. 8:**
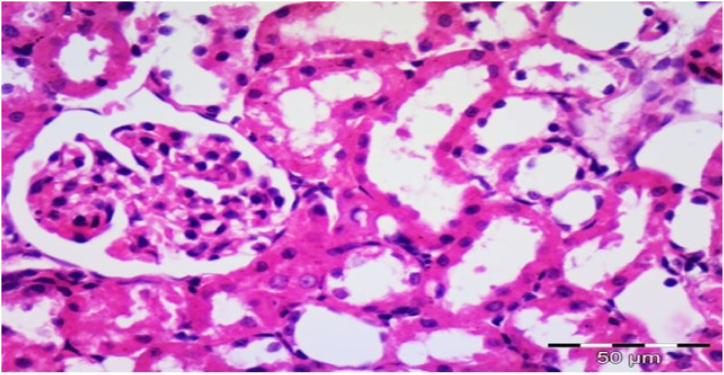
Photomicrograph of a section in the renal cortex of group II (received low protein diet) showing Malpighian renal corpuscle containing glomerulus without thickening of the glomerular basement membrane and no mesangial expansion (H&E, ×400).

In Group 2, Their renal cortex also showed Malpighian renal corpuscle containing glomerulus with no thickening of the glomerular basement membrane and no mesangial enlargement as mentioned at Fig. 8 and Table 3.

**Fig. 9:**
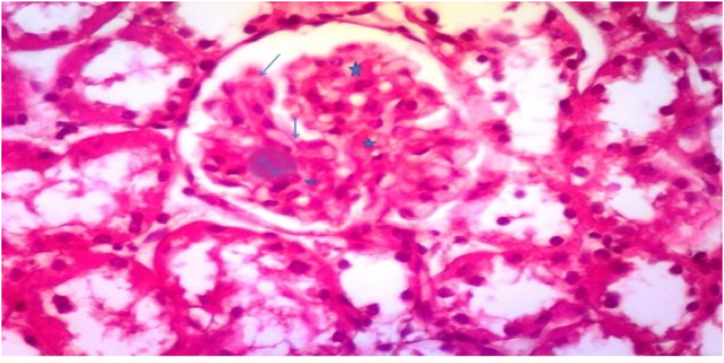
Photomicrograph of a section in the renal cortex of group III (Diabetic control received normal protein diet) showing marked diffuse expansion of mesangium (star), marked diffuse thickening of the glomerular basement membrane and nodular sclerosis (double headed arrow) (H&E, × 400).

In Group 3, Their renal cortex demonstrated substantial diffuse expansion of mesangium and marked diffuse thickening of the glomerular basement membrane, and nodular sclerosis as mentioned in Fig. 9 and Table 3.

**Fig. 10:**
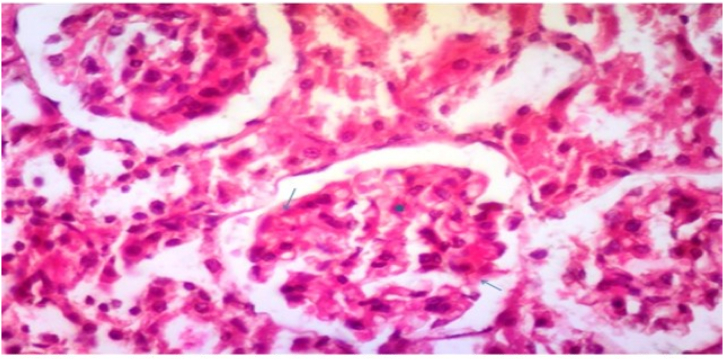
Photomicrograph of a section in the renal cortex of group IV (Diabetic received low protein diet) showing moderate diffuse expansion of mesangium (star), moderate diffuse thickening of the glomerular basement membrane and no nodular sclerosis (H&E, × 400).

In Group 4, They showed renal cortex with considerable diffuse expansion of mesangium and moderate diffuse thickening of the glomerular basement membrane, without nodular sclerosis as displayed in Fig. 10 and Table 3.

**Fig. 11:**
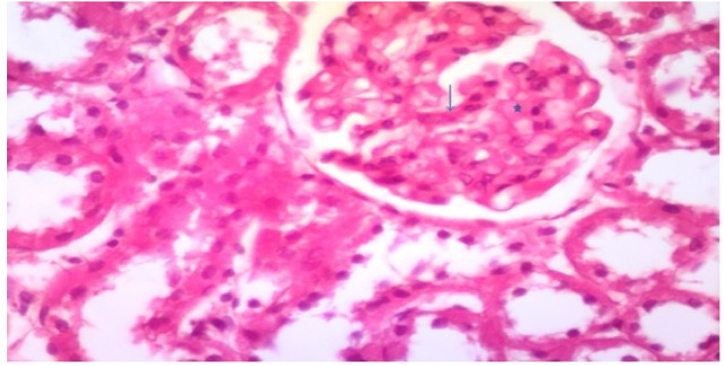
Photomicrograph of a section in the renal cortex of group V (Diabetic received α-keto amino acids with low protein diet) showing mild expansion of mesangium (star) and mild thickening of the glomerular basement membrane without nodular sclerosis (H&E, × 400).

In group 5, Their renal cortex exhibited minor mesangium growth and mild glomerular basement membrane thickening without nodular sclerosis as showed in Fig. 11 and Table 3.

**Fig. 12:**
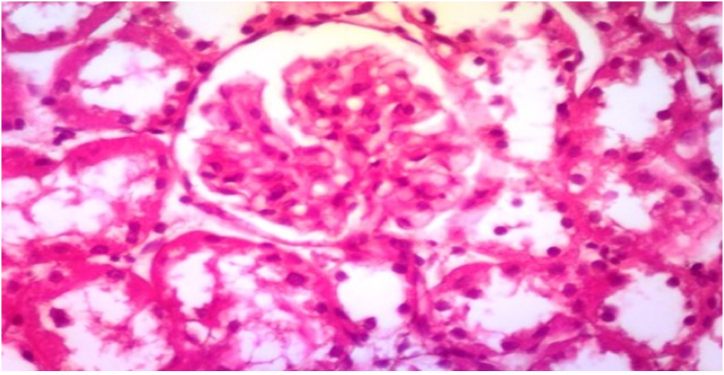
Photomicrograph of a section in the renal cortex of group VI (Diabetic received α-keto amino acids with free protein diet) showing normal Malpighian renal corpuscle containing glomerulus without thickening of the glomerular basement membrane, no mesangial expansion and no nodular sclerosis (H&E, ×400).

In Group 6, Their renal tissue showed normal Malpighian renal corpuscle containing glomerulus without thickening of the glomerular basement membrane, no mesangial expansion and no nodular sclerosis as mentioned in Fig. 12 and Table 3.

## 5 Discussion

Our aim in this work was to investigate the safety and efficacy of a protein-free diet KAA-supplemented diet in diabetic rats with CKD. The increase in CKD prevalence in diabetic patients even with tight control of DM makes us look for alternative ways to stop complications. Diet therapy is projected to be the safe alternative way to reduce the load on the kidneys and delay the occurrence of complications. The mainstay of conservative treatment in CKD is nutritional therapy, which aims to slow the course of clinical manifestations and may delay End-Stage Renal Disease (ESRD).

As a result, the protein-free diet was the foundation of our research. We found that the protein-free diet leads to an obvious increase in GFR and a decrease in urine protein with an improvement in blood glucose and insulin levels. There was also an improvement in kidney function and lipid profile.

Zhu. et al., reported that alpha-keto/amino acid supplementation decreases urine protein while the GFR remained relatively stable during the observation period with the nutritional status, inflammatory markers, and serum calcium, and phosphorus levels were not significantly affected [18].

In addition, Teplan et al.'s study sought to determine whether KAA supplementation provided further metabolic benefits in CKD patients receiving LPD. They found that the GFR, albumin and HDL levels were increased and the levels of urea, total cholesterol, LDL, and triglycerides also decreased with an associated decrease in proteinuria [19]. Studies by Solon-Otoda et al., and Kitada et al. have indicated that LPD had a reno-protective effect on CKD [20,21].

Our findings are corroborated by Rhee et al.'s meta-analysis, they found that in 16 controlled trials of a low-protein diet in CKD, preserved renal function and reduced ESRD, with no difference in the rate of malnutrition or protein-energy wasting between LPD and normal protein diet (NPD) [22,23]. In addition, studies by Ko and Kalantar, Khan et al. and Sakaguchi et al. found that 12 weeks of treatment with α-keto analogs of essential amino acids lead to a decline in blood glucose, urea, creatinine, and 24 h total urine protein. There was an increase in 24-h total urine volume and GFR. Even though, the low-protein meal had a larger carbohydrate content and was supplemented with ketoacids, the blood glucose level decreased. This suggests that, as Ko and Kalantar have noted, insulin sensitivity has been restored and glucose tolerance had improved [[24], [25], [26]].

This impact is explained by an increase in the production of insulin-like growth factor-1 (IGF-1) by the liver, which causes afferent arterioles in the glomeruli to expand and directly increase intraglomerular pressure. The renin-angiotensin system (RAS) is one further explanation for the beneficial effects of LPD in diabetic nephropathy as it is considered to be involved in most of the pathological processes that result in diabetic nephropathy.

Maintaining the nutritional status of the rats and preventing weight loss or muscle wasting due to protein deprivation was one of the important goals of the study. The results were promising, as the nutritional status of the rats was not affected by either weight loss or muscle wasting.

Rhee et al. found that a low-protein diet was related to better renal function maintenance and a lower rate of progression to ESRD. There was no difference, however, in the rate of malnutrition or protein-energy waste [23]. According to Bellizzi et al. during the diet period, body weight (BW) and muscle strength remained constant, and there were no changes to the nutritional markers or the multivariate analysis during the study [3]. The usage of KAA in conjunction with a very low protein diet reduces nitrogen intake. According to Sugahara, M. et al., KAA reduced proteinuria and improved renal function and nutritional condition in DM [6].

An increased carbohydrate content in LPD, along with a glucose homeostasis deficiency, may affect diabetes control in CKD. However, this does not occur in practice because a low-protein diet promotes insulin sensitivity and improves glucose tolerance. Insulin secretion is impaired in CKD due to urea levels, which decrease pancreatic insulin secretion.

## 6 Conclusion

Our study indicated that a free-protein diet containing KAA improves renal function, lowers blood glucose levels, maintains body weight, and does not worsen nutritional status in DM over time.

## CRediT authorship contribution statement

**Ahmed El-Sayed Nour El-Deen:** Writing – review & editing, Writing – original draft, Visualization, Validation, Supervision, Software, Resources, Project administration, Methodology, Investigation, Funding acquisition, Formal analysis, Data curation, Conceptualization. **Adel shallaby:** Writing – review & editing, Writing – original draft, Visualization, Validation, Supervision. **Abdel Kader Ibrahim:** Methodology, Investigation, Funding acquisition, Formal analysis, Data curation, Conceptualization. **Mohammed Abdel Aziz Mohammed:** Investigation, Formal analysis, Data curation. **Ahmad Taha:** Formal analysis, Data curation, Conceptualization. **Mohamed Zaeim Hafez Ahmed:** Formal analysis, Data curation, Conceptualization. **Muhammad Abdelbaeth Elfiky:** Formal analysis, Data curation. **Ahmed A. Abd El-Rhman:** Supervision, Software, Resources, Project administration, Methodology, Investigation. **Ahmed F. Abdel Ghany:** Software, Resources, Formal analysis, Data curation, Conceptualization. **Almoatazbellah Mahmoud Elsayed:** Visualization, Supervision, Methodology, Investigation, Funding acquisition, Formal analysis, Data curation, Conceptualization. **Ahmed Noaman Ali:** Software, Project administration, Methodology, Investigation, Funding acquisition, Formal analysis, Data curation, Conceptualization. **Ali Abdeslam:** Visualization, Validation, Software, Data curation.

## Consent for publication

The manuscript is original. It has not been published previously by any of the authors and even not under consideration in any other journal at the time of submission.

## Data and code availability

The data that support the findings of this study are available from the corresponding author upon reasonable request.

## Funding

The authors extend their appreciation to the 10.13039/501100021772Deanship of Scientific Research at 10.13039/501100010272Zarqa University for partial funding this work.

## Declaration of competing interest

The authors declare that they have no known competing financial interests or personal relationships that could have appeared to influence the work reported in this paper.
